# A Networked Sensor System for the Analysis of Plot-Scale Hydrology

**DOI:** 10.3390/s17030636

**Published:** 2017-03-20

**Authors:** German Villalba, Fernando Plaza, Xiaoyang Zhong, Tyler W. Davis, Miguel Navarro, Yimei Li, Thomas A. Slater, Yao Liang, Xu Liang

**Affiliations:** 1 Department of Civil and Environmental Engineering, University of Pittsburgh, 3700 O’Hara Street, 728 Benedum Engineering Hall, Pittsburgh, PA 15261, USA; gev5@pitt.edu (G.V.); fjp9@pitt.edu (F.P.); twd34@cornell.edu (T.W.D.); tas186@pitt.edu (T.A.S.); 2 Department of Computer and Information Science, Indiana University Purdue University, 723 West Michigan Street, SL 280, Indianapolis, IN 46202, USA; xiaozhon@umail.iu.edu (X.Z.); mignavar@iupui.edu (M.N.); liyim@imail.iu.edu (Y.L.); 3 Currently at USDA-ARS, Robert W. Holley Center for Agriculture and Health, 538 Tower Road, Ithaca, NY 14853, USA

**Keywords:** wireless sensor network, outdoor deployment, environment sensors, soil moisture, soil water potential, sap flow, MPS-2 sensor, TelosB mote, sensor board

## Abstract

This study presents the latest updates to the Audubon Society of Western Pennsylvania (ASWP) testbed, a $50,000 USD, 104-node outdoor multi-hop wireless sensor network (WSN). The network collects environmental data from over 240 sensors, including the EC-5, MPS-1 and MPS-2 soil moisture and soil water potential sensors and self-made sap flow sensors, across a heterogeneous deployment comprised of MICAz, IRIS and TelosB wireless motes. A low-cost sensor board and software driver was developed for communicating with the analog and digital sensors. Innovative techniques (e.g., balanced energy efficient routing and heterogeneous over-the-air mote reprogramming) maintained high success rates (>96%) and enabled effective software updating, throughout the large-scale heterogeneous WSN. The edaphic properties monitored by the network showed strong agreement with data logger measurements and were fitted to pedotransfer functions for estimating local soil hydraulic properties. Furthermore, sap flow measurements, scaled to tree stand transpiration, were found to be at or below potential evapotranspiration estimates. While outdoor WSNs still present numerous challenges, the ASWP testbed proves to be an effective and (relatively) low-cost environmental monitoring solution and represents a step towards developing a platform for monitoring and quantifying statistically relevant environmental parameters from large-scale network deployments.

## 1. Introduction

The sustainable condition of our freshwater resources partially depends on our understanding of the natural system in which it is cycled [1]. It has long been known that physically-based distributed hydrologic models require an understanding of the spatiotemporal variability of environmental data, which is difficult without an abundance of ground-based measurements for calibration and validation [2]. Soil moisture and transpiration play a fundamental role in the soil-atmosphere interactions and eco-hydrological processes. Moreover, the impacts of these and other hydrological parameters on regional hydrologic and climatologic conditions need permanent in situ measurements. Exploring the variability of soil moisture and transpiration at the plot scale and qualifying such measurements statistically can help improve estimates (including flux and storage components) of water budgets at the regional/watershed scale [3,4,5].

Ground-based measurements and monitoring of environmental variables have been impacted over the past decade by wireless sensor network (WSN) technology. Traditional data logging methods use cumbersome equipment, which is expensive to operate and inconvenient to maintain, that leads to its limited spatial coverage capabilities. Because of the high expense of sensors and data logging equipment, researchers are often forced to either forgo data loggers for high spatial density measurements with poor temporal resolutions (i.e., hand measurements) or obtain high temporal resolution at a limited number of strategically located data loggers.

Small, inexpensive, wireless monitoring devices are pervading beyond networking and communications research fields. These devices are providing scalable, high resolution data at a declining cost [6,7] and have found applications in a variety of environmental monitoring fields, including: habitat monitoring [8,9,10], microclimate monitoring [11,12], seismology [13,14], understory sunlight studies [15], agriculture [16,17,18,19,20,21,22,23,24], ecology [25,26,27,28] and hydrology [29,30,31,32,33]. High-resolution sensor networks of plot-scale hydrology is a growing application for WSNs due, in part, to the increasing demand for calibrating and characterizing sub-grid variability of airborne and space-borne measurements [34,35].

A long-term (over six years) WSN has been measuring edaphic (e.g., moisture, water potential and temperature) and arboreal (i.e., xylem sap flow) hydrological properties in a forested nature reserve at the Audubon Society of Western Pennsylvania (ASWP) [32]. The original motivation of the ASWP network was to determine the feasibility of using WSNs to continuously and reliably collect hydrological data under natural outdoor conditions. Following the successful deployment of the network, which has been running on TinyOS 2.1.2 [36] and CTP [37], comes a new stage of research, starting in 2015, aimed towards network expansion and improvement of data collection and processing.

The novelty of this WSN study includes: (1) a data acquisition board design and software driver for integrating digital environmental sensors into the wireless hardware platform; (2) an energy efficient and balanced routing protocol CTP + EER [38]; and (3) a heterogeneous over-the-air mote-reprogramming tool. Furthermore, our WSN enables the study of the following: (1) assessment of the quality of the data collected from soil moisture, soil water potential and soil temperature sensors attached to WSN nodes using a specially designed sensor board; (2) retrieval of high quality sap flow measurements from our lab-made [39] Granier-style [40,41] sap flow sensors; (3) the application of the collected data to: (a) estimate soil hydraulic properties; (b) calculate transpiration based on sap flow; and (c) explore spatiotemporal patterns of soil moisture and soil water potential; and (4) evaluation of the utility of WSNs for environmental monitoring applications. To the best of our knowledge, this is the first comprehensive study to address these important questions from a single network perspective.

## 2. Materials and Methods

### 2.1. Equipment

**Figure 1:**
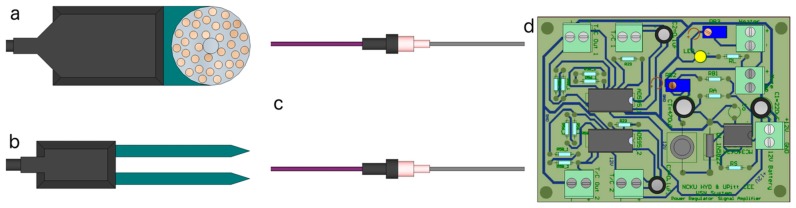
Schematics of the environmental sensors deployed at the ASWP network, including the (**a**) Decagon Devices MPS-1/MPS-2 water potential (WP) sensor; (**b**) Decagon Devices EC-5 soil moisture (SM) sensor; (**c**) thermometric sap flow sensor probes; and (**d**) sap flow sensor circuit.

Three types of external sensors were used throughout the study site. The first two are the MPS-1 and EC-5 sensors (Decagon Devices, Pullman, WA, USA) which provide measurements of matric water potential (WP) and volumetric soil moisture (SM) (Figure 1a,b, respectively). The Decagon Devices MPS-2 digital sensor [42], which provides measurements of soil temperature in addition to WP, is also deployed within the network due to the discontinuation of the MPS-1 sensor. The third sensor is a pair of Granier-style thermal dissipation (constant heat) sap flow sensor probes (Figure 1c), which were made and calibrated following [39]. The sap flow probes are connected to the mote’s sensor board via a control circuit (Figure 1d) to amplify and condition the thermocouple response voltage as shown in [43]. The sap flow control circuit is operated by a 12 V lead-acid battery to accommodate the additional power requirements of the thermal dissipation method.

#### 2.1.1. Initial Equipment (MICAz, IRIS, MDA300)

The network was built on an existing investment in WSN hardware, manufactured by Crossbow Technology (now MEMSIC, Inc., Andover, MA, USA), which includes the MPR2400 (MICAz) and XM2110 (IRIS) processor and radio boards (i.e., wireless motes) and the MDA300 sensor board. The wireless motes are powered using rechargeable nickel-metal hydride (NiMH) batteries (size AA and D). The batteries, after recharging, are sorted based on their rested voltages to avoid deploying partially charged or uncharged batteries (see Section 3.5.1 for details). The data collection software, including data sampling and packet routing, is developed based on the state-of-the-art open-source WSN platform TinyOS [36].

#### 2.1.2. Updated Equipment (TelosB Motes and Custom Sensor Board)

Starting in 2015, the wireless motes have gradually been updated to the CM5000-SMA (TelosB) by Advanticsys (Madrid, Spain) with our specially designed sensor board for data acquisition, forming a heterogeneous WSN consisting of MICAz, IRIS and TelosB motes. TelosB motes incorporate the 2.4 GHz CC2420 transceiver and the MSP430 microcontroller with 10 KB of RAM and provide a 16-pin expansion interface to connect external sensors. For the sensors described above, an excitation voltage is required before a reading. In the standard method, general-purpose input/output (GPIO) pins are used as excitation pins and ADC pins are used to gather sensor readings; however, when powering sensors directly using GPIO pins, the excitation voltage is unstable and can change under different workloads and battery levels. Since analog sensor readings are proportional to the excitation voltage, the readings might be inconsistent even in the same environment.

**Figure 2:**
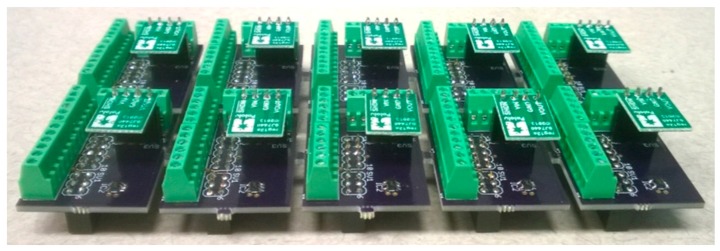
Custom sensor boards for TelosB motes.

To address this problem, a novel custom sensor board was designed for TelosB motes (Figure 2) using voltage regulators to provide a stable excitation voltage. In the literature, TelosB acquisition boards are usually designed for specific sensors, such as motion sensors [44,45,46], physiological sensors [46], and SM sensors [47]. In contrast, our design is a generic sensor board for the TelosB mote. Our sensor board has two distinguishing features. First, it has ADC channels for analog sensor output and a UART channel for digital sensor output. Second, it provides two levels of stable excitation voltage that enables different combinations of external sensors to be attached based on the application configuration, which is essential in heterogeneous networks, such as the ASWP network.

The sensor board is attached to the TelosB mote’s 16-pin expansion, providing screw wire connectors, ADC channels, UART0 serial port, and two excitation voltages. Analog sensors, such as EC-5 and MPS-1, can be attached to ADC channels and powered through a 2.5 V excitation voltage, obtained by using a TLV70025 voltage regulator (Texas Instrument, Dallas, TX, USA). Digital sensors can be connected to the UART0 serial port, such as the MPS-2, which generates a byte stream representing ASCII characters as its sensor readings. The MPS-2 requires an excitation voltage between 3.6 V and 15.0 V. A 5 V voltage booster (U1V11F5, Pololu, Las Vegas, NV, USA) is included in the custom sensor board to provide a 5.0 V excitation voltage from the 3.6 V nominal battery supply to power the MPS-2 sensors.

#### 2.1.3. Enclosures

**Figure 3:**
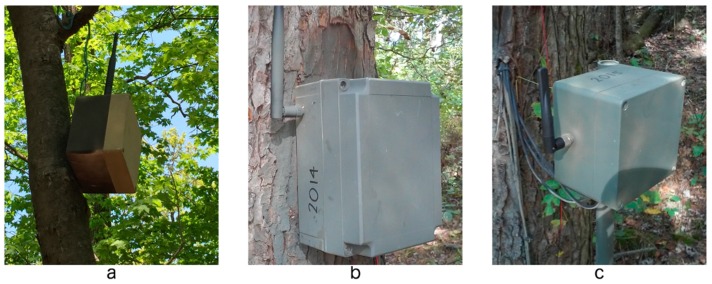
Examples of node types and their enclosures in the ASWP network, including (**a**) relay nodes hanging from a tree branch; (**b**) sap flow node mounted to the side of a tree; and (**c**) soil sensor node mounted to a PVC pipe.

The wireless motes and all necessary electronics are housed inside water-tight polycarbonate high-impact enclosures, connected to an external omni-directional high-gain (4.9 dBi) antenna, and are discretely hung from tree limbs, attached to PVC posts, or mounted to the sides of trees (Figure 3). Motes with sap flow sensors deployed during the 2015 and 2016 growing seasons are powered by the sap flow control circuit’s power regulator; therefore, these motes did not require the AA or D rechargeable batteries.

### 2.2. Mote Application

The mote application was developed in TinyOS 2.1.2 [36] with an adoption of the newly developed routing protocol CTP + EER [38]. TinyOS is the most widely used WSN operating system and is found in 60% of WSN deployments [48,49]. Owing to its popularity, TinyOS has a larger community, better documentation, and well-tested drivers and protocols compared to other WSN operating system alternatives [48].

CTP + EER is an efficient and balanced routing protocol that extends CTP [37]. CTP is the de facto standard collection routing protocol in TinyOS, in which each mote finds the best route to the sink (i.e., base station). Unfortunately, with this protocol, network data traffic tends to concentrate on a few specific motes that provide the best routes within the network. Thus, these motes experience heavy congestion and deplete their batteries faster than their neighbors. In contrast, CTP + EER, while maintaining the best primary route within the network, also allows motes to select suboptimal routes from a parent set; therefore, it can reduce the data traffic at the busiest motes and provide better overall energy efficiency and balance. CTP + EER has been evaluated though analytical modeling, simulations, and testbed experiments. Compared with CTP, CPT + EER achieves better packet reception ratio, load balance, and energy efficiency. Please see [38] (and the references herein) for more details.

**Figure 4:**
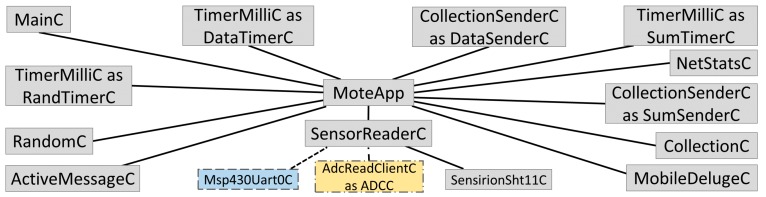
Mote application architecture.

The wireless motes form a multi-hop collection network operating in asynchronous low-power listening (LPL) [50]. The logical architecture of the application is presented in Figure 4. Each node uses CTP + EER to deliver two types of packets: data packets (DataSenderC) and summary packets (SumSenderC). Data packets are periodically sampled at a base interval of 30 min (DataTimerC). Randomness is added to each mote to avoid bursty network traffic (RandTimerC). Summary packets enable efficient network diagnosis (NetStatsC) and are generated every two hours (SumTimerC), which includes the network statistics such as retransmission, dropped packets, and information about the routing control traffic.

The mote application was adjusted based on the sensor types attached to each individual mote. There are two types of nodes: relay nodes and sensor nodes. Relay nodes have no external sensors and are used at advantageous locations to improve communication throughout the network (e.g., hanging in trees as shown in Figure 3a). Sensor nodes are nodes with external environmental sensors (e.g., SM, WP, soil temperature or sap flow). Sensor nodes also participate in network routing and packet forwarding in data collection. Relay nodes only have temperature and humidity sensors (Sht11C, Sensirion, Zürich, Switzerland) [51]. Sensor nodes have analog sensors attached that utilize the ADC channels. In addition, some sensor nodes with TelosB motes also have a digital sensor that communicates through the UART0 serial port (Msp430Uart0C).

To facilitate the reprogramming of motes that are deployed in difficult-to-access enclosures, an over-the-air mobile mote-reprogramming tool was utilized such that direct access to the mote hardware was not necessary. While many over-the-air programming approaches have been proposed for WSNs, none of them apply to heterogeneous and low power WSNs [52,53]. Our novel mobile mote reprogramming tool, MobileDeluge [52] (see Section 3.5.4), was developed to overcome this limitation.

### 2.3. Deployment

**Figure 5:**
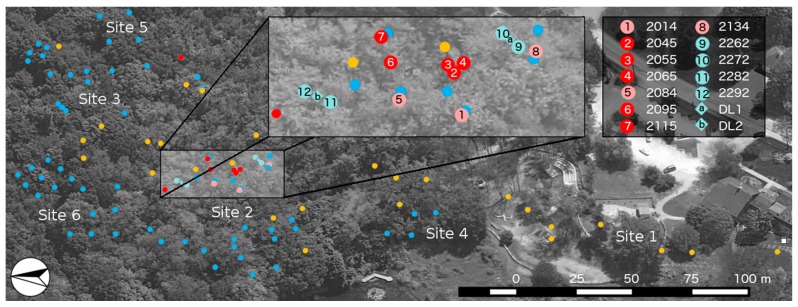
Map of the six sites of the ASWP testbed (October 2016 configuration). Relay nodes are represented as yellow circles, sap flow nodes are represented as red circles (the three pink circles in site 2 are used in this analysis), soil sensor nodes are represented as dark blue circles, and the base station is represented as a white square. The data loggers used for validation (i.e., DL1 and DL2) are shown as light blue diamonds and their corresponding nodes as light blue circles. The four-digit node numbers referenced in the analysis are indicated in the zoomed region of site 2.

The ASWP network was initially formed in 2010, which culminated in 2014 as a 52-node deployment located over five sites as described in [32]. The network has since doubled in size (i.e., 104 nodes) due to a 36-node addition during the summer of 2015 and a 16-node addition during the summer of 2016. The study area now includes six sites, of which five are designated areas for environmental monitoring. Figure 5 shows the locations of the relay (yellow circles) and sensor (red and blue circles) nodes throughout the six sites of the deployment. The base station (white square) is located in an office window of the nature reserve building in site 1.

During the initial four years of the network deployment, all sensor nodes consisted of one MPS-1 and two EC-5 sensors. A subset of these sensor nodes was also outfitted with a sap flow sensor and a control circuit (operated during the growing seasons). Since the summer of 2014, sensor nodes have been divided into two classes: soil sensor and sap flow sensor nodes.

The soil sensor nodes include two EC-5 sensors and one MPS-2 sensor (replacing the MPS-1). One of the two SM sensors is co-located with the WP sensor at a depth of 30 cm, separated by enough distance such that the measurements of one would not interfere with the other. In the same hole, the second EC-5 sensor is placed at a depth of 10 cm. Best efforts were made to avoid rocks and tree roots during sensor installation. Holes are drilled into the bottom of the sensor node enclosures to allow the sensor wires to be connected to the sensor board (Figure 3c).

The sap flow sensor nodes are equipped with one set of sap flow probes connected to a sap flow control circuit. The sap flow node enclosure houses all the wireless and sensor electronics (Figure 3b). Two holes are drilled through the back wall of the enclosure (i.e., side facing the tree), spaced approximately 10 cm apart for seating the sap flow probes into the tree. Before attaching the enclosures to the tree, a portion of the tree bark is stripped away to create a flat surface for the enclosure box and to increase the penetration depth of the probes into the tree’s active xylem. Enclosures are attached to the trees using wood screws with the two enclosure holes aligned parallel to the tree’s vertical growth axis. Once enclosures are attached to the tree, pilot holes for the sensor probes are drilled horizontally into the tree at the locations of the two enclosure holes and are given a prophylactic treatment of hydrogen peroxide. To aid in installation and removal while improving the thermal conductivity with the tree xylem, the probe needles are coated with petroleum jelly. Once inserted into the tree, the probes are fixed inside the enclosure using a silicone adhesive, which also prevents water from entering the enclosure through the holes.

As a means of validating the WSN soil sensor measurements, two Decagon Devices EM50 data loggers were deployed during the summer of 2016 along the hill slope that stretches from the bottom of site 2 to the top of site 3 (i.e., light blue diamonds in Figure 5). Accompanying the data loggers are four additional nodes (i.e., light blue circles in Figure 5), two surrounding each data logger. The validation data loggers and nodes are located at approximately the midpoint of the hill (i.e., nodes 2282, 2292 and data logger DL2) and close to the lower part of the hill (i.e., nodes 2262, 2272 and data logger DL2) in site 2, respectively. Each validation node is connected to three soil sensors and each data logger is connected to five soil sensors in such a way that five out of the six node sensors are matched with a data logger sensor (i.e., the same location, sensor type and installation depth). The sensor type and installation depth are the same as the other soil sensor nodes in the network.

### 2.4. Calibration of Soil Moisture Measurements

The SM raw data is collected as a voltage (mV) from the EC-5 sensor attached to a sensor board via an ADC (analog-to-digital-converter). The raw data needs to be converted to SM using a conversion equation. Typically, the conversion equation is presented as a linear equation *θ* = *c*1 × ADC + *c*0, where ADC is the raw voltage output (in mV) from the EC-5 sensor and *c*1 and *c*0 are the slope and intercept of the fitted linear regression model, respectively. The standard coefficients for non-Decagon data loggers at an excitation of 2.5 V for mineral soils, are *c*1 = 0.00119 and *c*0 = −0.401.

Estimates of SM based on the standard equation showed a bias towards drier conditions. To increase the accuracy of the estimation, the field data collected at the validation locations were calibrated by a linear regression using the ordinary least squares (OLS) method. The targets values are from the validation data logger and the input is the raw data (i.e., ADC in mV from EC-5 sensor) from the validation nodes. The EC-5 sensors were calibrated using an intercept of −0.360 and −0.367 for depths of 10 and 30 cm, respectively, and slopes of 0.0011 and 0.0012 for depths of 10 and 30 cm, respectively. The slope values were found to be similar to the standard value (i.e., 0.00119), while the intercept values are lower in magnitude.

### 2.5. Hydraulic Properties from Soil Moisture Measurements

One of the benefits of in situ plot-scale hydrology studies is the ability to estimate the hydraulic properties that govern the region. These hydraulic properties are important in characterizing a region with estimates to pedotransfer function (PTF) parameters that are utilized by hydrologic models to predict soil water retention properties based on available soil survey data [54,55]. In this work, two PTFs are examined. The first PTF is the Clapp-Hornberger equation, which is given by the following power curve [56]:

(1) $$s={\left(\frac{\psi }{\psi_{s}}\right)}^{\frac{-1}{b}},$$

where ψ is the hydraulic conductivity, $\psi_{s}$ is the hydraulic conductivity at saturation, and $s=\theta /\theta_{s}$ is the soil wetness, a ratio of the SM, θ, to the saturated SM, $\theta_{s}$ (i.e., total porosity).

The second PTF is the van Genuchten equation, given by the following expression [57]:

(2) $$\frac{\theta -\theta_{r}}{\theta_{s}-\theta_{r}}={\left[1+{\left(\alpha |\psi |\right)}^{n}\right]}^{-m},$$

where $\theta_{r}$ is the residual SM, m=1−1/n, and n and α are fitting parameters. Assuming, for simplicity, that $\theta_{r}$ is zero, the left-hand side of Equation (2) may be expressed in terms of s, such that:

(3) $$s={\left[1+{\left(\alpha |\psi |\right)}^{n}\right]}^{-m},$$

Field measurements of θ and ψ are used for fitting values of $\psi_{s}$ and b in Equation (1) and α and n in Equation (3). The value for $\theta_{s}$ was determined experimentally based on the following methodology. A soil core sample was taken at a depth of 10 cm and 30 cm at the two node locations surrounding the validation data loggers (Figure 5). The soil samples were weighted under field conditions and then dried in an oven at 100 °C for 48 h before being weighted again. The bulk density was calculated as the dry weight divided by the volume of the soil sample core. The porosity was calculated as one minus the bulk density divided by the particle density, which was assumed as 2.65 g·cm^−3^.

### 2.6. Transpiration Calculations from Sap Flow Measurements

In this study, the xylem sap flow (i.e., the velocity of the water being transported through the active sapwood of the tree) is calculated using an empirical equation based on daily temperature differences between a pair of heated and reference temperature probes [41,58]:

(4) $$Q_{s}=0.000119\times {\left(\frac{\Delta T_{0}-\Delta T}{\Delta T}\right)}^{1.231},$$

where ΔT is the temperature difference between the upper and lower probes (°C); $\Delta T_{0}$ is the maximum daily value of ΔT (i.e., zero sap flow) (°C); and $Q_{s}$ is the sap flux density (m^3^·m^−2^·s^−1^). Calculated quantities of $Q_{s}$ are converted to transpiration based on the following equation [59,60,61,62]:

(5) $$\tau =Q_{s}\left(\frac{A_{s}}{A_{G}}\right),$$

where τ is the rate of transpiration (m·s^−1^); $A_{s}$ is the tree sapwood area (m^2^); and $A_{G}$ is the ground area (m^2^). The ratio $A_{s}/A_{G}$ depends on the study site and is indicative of the tree density and the predominant tree species. It has been shown that this ratio can be as low as 1 m^2^·ha^−1^ [63] and reach values as high as 25 m^2^·ha^−1^ [61] or 40 m^2^·ha^−1^ [64]. Estimates of $A_{s}$ may be determined empirically, such as by the following allometric equation [65]:

(6) $$A_{s}=B_{0}\cdot d^{B_{1}},$$

where d is the measured tree diameter at breast height (cm) and $B_{0}$ and $B_{1}$ are species-specific coefficients determined by regression techniques.

In a 2010 sap flow study at the ASWP site [66], 22 trees were surveyed to identify their species, take measurements of d, and estimate $A_{s}$ from three core samples taken at breast height. Based on the results of that study, only the silver maple (*Acer saccharinum*) trees, predominantly in site 2, produced measurable sap flow quantities.

**Figure 6:**
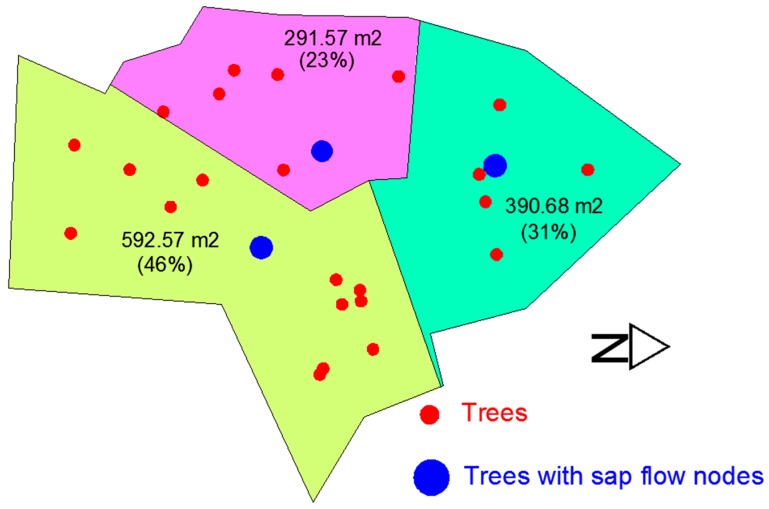
Field survey area within site 2 for the determination of a representative *A_S_/A_G_* ratio.

In 2016, another survey was conducted to estimate $A_{s}/A_{G}$ for the silver maples in site 2. Figure 6 shows the surveyed area in site 2 for the $A_{s}/A_{G}$ ratio estimation (approximately 1300 m^2^) where three sap flow nodes (2014, 2084 and 2134) are located (pink circles in Figure 5). To establish the boundary of the surveyed area, first, a preliminary perimeter was defined using trees located around the sap flow nodes. Then, this perimeter was displaced 5.33 m, which is the approximate mean distance between neighboring trees. The area of influence for each sap flow node was established using the Thiessen polygons criterion (i.e., the colored regions in Figure 6). These regions were used to calculate one $A_{s}/A_{G}$ ratio for each node according to the number and diameter of trees within their area of influence. The diameters at breast height of 25 trees, including the three trees with sap flow sensors, were measured and the sapwood areas, calculated using Equation (6), are compared with the measurements made in 2010.

### 2.7. Geostatistical Analysis of Soil Moisture and Soil Water Potential: Spatiotemporal Trends

SM and WP are important variables in the water cycle within climate systems. Thus, the quantitative estimation of these parameters is fundamental for application fields such as weather forecast, hydrology and watershed management [67]. SM, for instance, usually shows strong spatial variability due to physical and geographic characteristics of the environment (e.g., topography, soil type, vegetation coverage) [68,69]. Surface interpolation methods such as Kriging are widely used to assess the spatial characteristics of hydrologic variables [67,70,71,72,73,74,75].

The Ordinary Kriging (OK) interpolation method is the most widely used geostatistical interpolation technique and is acknowledged as the standard approach for surface interpolation [35,65,70,76,77,78,79,80,81]. OK assumes that the distance or direction between sample points reflects a spatial correlation that can be used to explain variation in the surface. The spatial dependence is expressed by a semi-variogram. This method is appropriate when it is known that there is a spatially correlated distance or directional bias in the data, as is with SM and WP. The OK estimation equation is given by the following:

(7) $$Z_{\left(s_{0}\right)}=\sum_{i=0}^{n}\lambda_{i}\cdot Z_{\left(s_{i}\right)},$$

where $Z_{\left(s_{i}\right)}$ is the measured value at the *i*-th location, *λ_i_* is an unknown weight for the measured value at the *i*-th location, $Z_{\left(s_{i}\right)}$ is the predicted value at the prediction location *S*_0_, and *n* is the number of measured values. The weight, *λ_i_*, depends on a fitted model to the measured points, the distance to the prediction location, and the spatial relationships among the measured values around the prediction location. In this study, an OK interpolation method with a spherical semi-variogram model is used to estimate the spatiotemporal trends of SM and WP, since this model has been found to satisfactorily represent the spatial dependence in previous studies [67,70,76,82,83,84]. The root mean square error (RMSE) is used to assess the performance of the selected interpolation method.

## 3. Results and Discussion

### 3.1. Data Quality Assessment of WSN Sensors

The data quality assessment of the WSN soil sensors was performed at the validation locations in site 2 where data logger measurements were accompanied by sensor node measurements at the same time and location. Only the validation results at the midpoint location of site 2 are presented here.

**Figure 7:**
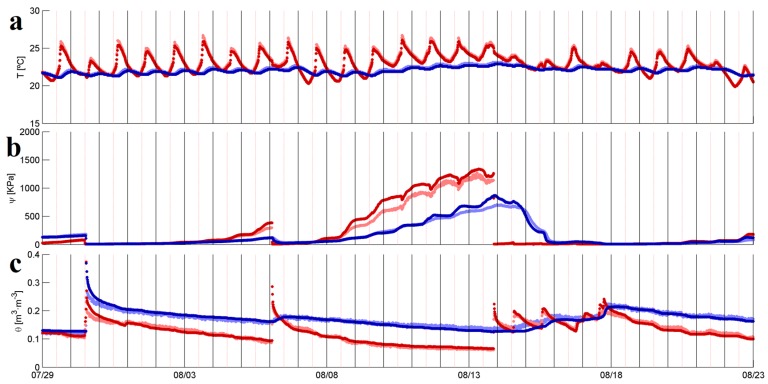
Comparison of (**a**) soil temperature, T in Celsius degrees; (**b**) matric water potential (WP), ψ in kPa; and (**c**) volumetric soil moisture (SM), θ in m^3^·m^−3^ data collected by a data logger (DL2) and wireless nodes (2282 and 2292) from the ASWP network between 29 July and 23 August 2016. The variable at a depth of 10 cm is shown in dark red for the data logger and light red for the nodes. The variable at a depth of 30 cm is shown in dark blue for the data logger and in light blue for the nodes.

Figure 7 shows the comparison results of soil temperature, T (Figure 7a), WP, ψ (Figure 7b), and SM, θ (Figure 7c), for the time period between 29 July and 23 August 2016 for the data logger DL2 and the nodes 2282 and 2292 at two depths near the midpoint of the hill in site 2. The soil temperature measurements (based on the MPS-2) from the WSN peak slightly higher than the data logger measurements (light lines in Figure 7a) and are indistinguishable at the validation location at the bottom of the hill (not shown). The WP measurements (also based on the MPS-2) are slightly lower at both depths from the WSN compared to the data logger (Figure 7b). The SM measurements (based on the EC-5) are nearly indistinguishable between the WSN nodes and the data logger.

### 3.2. Hydraulic Properties Estimation

**Figure 8:**
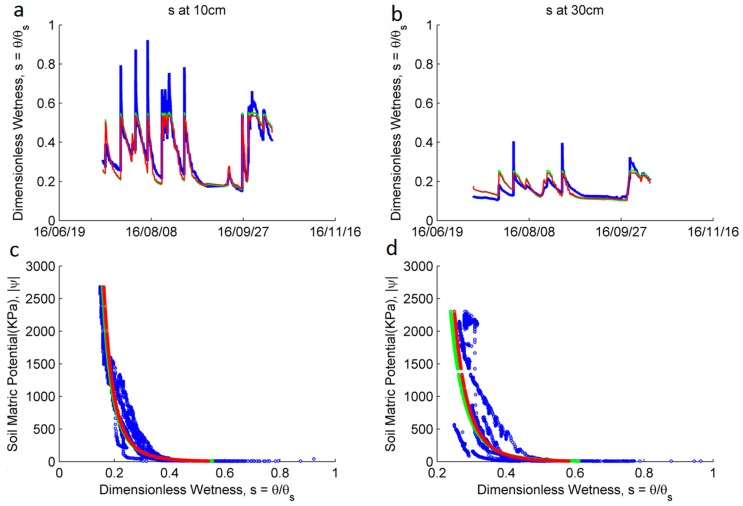
Estimation of the soil hydraulic parameters from data at the location close to the middle of the hill in site 2 (i.e., Nodes 2282 and 2292, and data logger DL2). Wetness, *s*, in blue; Estimated wetness from measured matric water potential (WP) using the Clapp and Hornberger, and van Genuchten equations, in green and red, respectively. (**a**) Comparison of the wetness, *s*, time series, at a depth of 10 cm; (**b**) Same as part a, for a depth of 30 cm; (**c**) Relationship between the soil wetness, *s*, and the absolute value of the WP, in kPa, |ψ| at a depth of 10 cm. The fitted Clapp and Hornberger equation is shown in green and the fitted van Genuchten equation in red; (**d**) Same as part c, for a depth of 30 cm.

Figure 8 shows the empirical relationship between the SM, θ, and the absolute value of the WP, in kPa, |ψ| at a depth of 10 cm and 30 cm for the location close to the middle of the hill (i.e., nodes 2282, 2292 and data logger DL2) in site 2. The fitted equations lead to similar results for both equations. At a depth of 10 cm the fitted parameter for the Clapp and Hornberger equation are: $\psi_{s}$ = 0.658 kPa and b = 4.49; For the van Genuchten equation: n = 1.215 and α = 1.808. The porosity value $\theta_{s}$ = 0.31 was calculated from a soil core taken in the same location. For the depth of 30 cm, the parameters for the Clapp and Hornberger equation are $\psi_{s}$ = 0.521 kPa and b = 5.87; For the van Genuchten equation: n = 1.154 and α = 3.51. The porosity value $\theta_{s}$ = 0.42 was calculated from a soil core taken in the same location. Table 1 summarizes these results, including the location close to the lower part of the hill (i.e., Node 2262, 2272 and Data logger DL1). The fitted equations were evaluated using the Nash-Sutcliffe efficiency (NSE) [85].

### 3.3. Sap Flow Data and Transpiration Estimation

#### 3.3.1. Sap Flow Time Series

**Figure 9:**
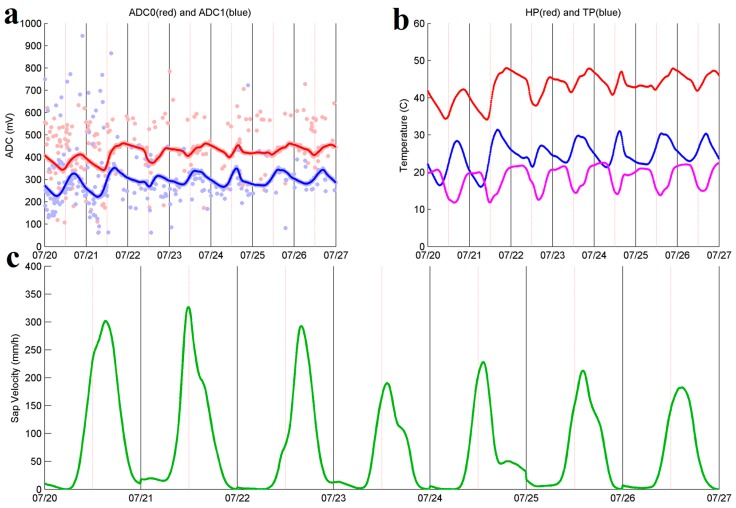
Sap flow results for node 2084 between 20 and 27 July 2016. (**a**) Raw voltages (i.e., ADC in mV) from the HP (red scatter plot) and TP (blue scatter plot) between 0 and 1000, and smoothed plot for the HP and TP in red and blue, respectively; (**b**) Filtered and smoothed temperatures for the HP (red) and TP (blue) from the raw voltages ADC0 and ADC1 respectively, difference in temperature (HP-TP) in Celsius degrees (magenta); (**c**) Sap flow time series (mm/h).

Figure 9 shows the results of the sap flow collected by one WSN node (i.e., node 2084) the week between 20 and 27 July 2016. Figure 9a shows the raw voltage measurements between 0 and 1000 mV from the sap flow probes collected by the same node; however, measurement noise produced raw voltage readings as high as 1500 mV (not shown).

To perform an accurate estimation of the voltages for each probe, a robust weighted local regression [86] is used. The robust weighted local regression smooths the raw data and it is not affected by a relatively small number of outliers. The smoothed results are shown as red and blue lines in Figure 9a for the heater probe (HP) and the temperature probe (TP), respectively. Figure 9b shows the temperature conversions from the smoothed raw measurements based on the individual calibrations for the HP and TP in red and blue, respectively. The difference in temperature between the HP and TP (i.e., ΔT=HP−TP) in Celsius degrees is shown in Figure 9b, in magenta. Finally, Figure 9c shows the resulting sap velocity based on Equation (4).

#### 3.3.2. Transpiration Estimates from Sap Flow Measurements

Table 2 shows a comparison between the $A_{s}$ estimates of monitored silver maple trees in site 2 (Figure 5 for locations) and modeled sapwood area, ${\hat{A}}_{s}$, based on Equation (6), for which the same values of d were used from the field survey. The coefficients $B_{0}$ and $B_{1}$ were selected according to the tree species [61]. It is observed that the values of ${\hat{A}}_{s}$ are similar to the field estimates (RMSE = 78.6 cm^2^); therefore, Equation (6) was used to calculate the sapwood area of the trees located inside the 1300 m^2^ survey area.

Table 3 shows the results for the $A_{S}/A_{G}$ ratios for the three sap flow nodes based on their regions of interest within the survey area (Figure 6).

**Figure 10:**
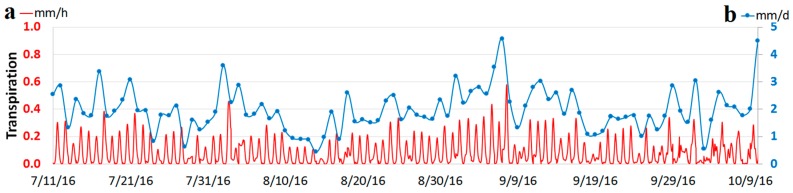
Transpiration (τ) calculations in the ASWP site based on the measurements in nodes 2014, 2034 and 2134, from 11 July 2016 (7/11/16) to 11 October 2016 (10/11/16). (**a**) Transpiration rates in mm/h based on a 10-min interval; (**b**) Transpiration rates in mm/day based on a 24-h interval.

Figure 10 shows the transpiration (τ) calculations using Equation (5) for the sap flow measurements from nodes 2014, 2084 and 2134 based on a mean-weighted $A_{S}/A_{G}$ ratio of 12.06 m^2^·ha^−1^ from Table 3. The selected time period (i.e., from July to October) represents the time of year when, on average, most of the evapotranspiration occurs around the study site [87]. Figure 10a shows τ, in mm/h, every 10 min, which corresponds to the sap flow sensor’s sampling interval. The peaks in Figure 10a represent a time close to noon on each day. Despite having few noticeable high peaks, τ is mostly within the range 0.2–0.4 mm/h.

Integrating the hourly τ rates in Figure 10a to monthly totals yields 40.8 mm from 11 to 31 July, 55.2 mm from 1 to 31 August, 65.4 mm from 1 to 30 September, and, 30.7 mm from 1 to 11 October. According to the NRCC, the monthly average potential evapotranspiration (PET) estimates for the greater Pittsburgh area are 110.7 mm for July, 96.3 mm for August, 66.3 mm for September and 39.12 for October. Considering the average values from NRCC as a reference, these results suggest that there could be an overestimation of the τ during September and October, since monthly τ calculated for September is very close to the estimated average PET (i.e., 65.4 mm compared to 66.3 mm) and the monthly τ calculated for 11 days of October (i.e., 30.7 mm) are projected to be higher than the estimated average PET (i.e., 39.12 mm). Besides two noticeable peaks in τ during these two months (i.e., 7 September and 10 October in Figure 10a), the remaining values are consistently higher than in the previous months (i.e., July and August). This suggests that for these two months, the τ rates were higher than average and there is not an overestimation of the transpiration. However, since the $A_{S}/A_{G}$ ratio is a major factor controlling τ rates, an extension of the survey area can be considered for a better estimation.

Figure 10b shows the daily τ values, which is consistent with previous studies that have similar geographic and climatic characteristics to the ASWP site [60,61,88,89]. The maximum, minimum and average values of daily τ during the specified period of time are 4.49 mm, 0.44 mm and 2.01 mm, respectively.

### 3.4. Exploration of Soil Moisture and Soil Water Potential: Spatiotemporal Trends

Determining and explaining the temporal and spatial hydrological patterns is one of the major challenges in the hydrological sciences, since the factors that control these patterns behave in a nonlinear way [35]. In this study, a spatial analysis was performed in order to show the variability of SM and WP.

**Figure 11:**
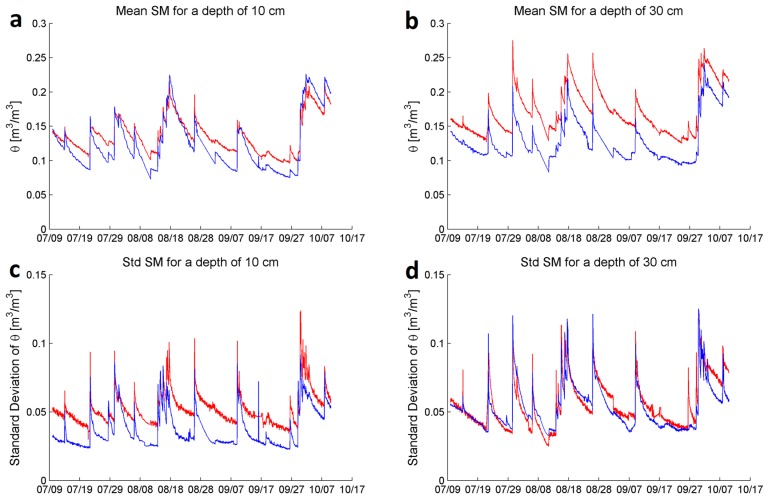
Comparison of the mean and standard deviation of volumetric soil moisture (SM), θ in m^3^·m^−3^ at sites 2 and 6 in red and blue, respectively, in the ASWP WSN testbed between 10 July and 10 October 2016. (**a**) Mean SM at a depth of 10 cm; (**b**) Mean SM at a depth of 30 cm; (**c**) Standard deviation of the SM at a depth of 10 cm; (**d**) Standard deviation of the SM at a depth of 30 cm.

Figure 11 shows the time series of mean SM and its standard deviation at two depths (10 and 30 cm) for sites 2 and 6. The mean and standard deviation were calculated using hourly time series for each node in sites 2 and 6. Site 6 is characterized by a steep hill slope, while the slope is moderate for site 2. Figure 11a,b show that the mean SM at both depths in site 2 is generally higher than that at site 6. The SM is especially higher at 30 cm (Figure 11b). Figure 11c,d show the standard deviation at sites 2 and 6, at 10 and 30 cm, respectively. It is shown that site 2 has a higher standard deviation than site 6, especially at 10 cm.

Figure 11 illustrates that, due to the presence of significant heterogeneity within a small spatial scale (e.g., the two sites are only a few meters away from each other), individual measurements (e.g., SM in this case) from the nearby locations can be quite different. To capture the variability of SM within a small spatial scale would require many sensors within an area of study. The traditional approach of connecting one or a limited number of sensors to a single data logger is not practical as it would require a large number of data loggers that would make the installation and maintenance cost prohibitive. In contrast, the WSN approach, together with the network protocol used here, makes such applications feasible as the cost is relatively low and data processing is centralized and simplified.

With these dense SM measurements, one can investigate scientific questions such as, how would the spatial variability of SM (both horizontally and vertically) affect evapotranspiration? To what extent, would the lack of knowledge of the spatial variability of SM lead to significant errors on hydrological modeling results? In addition, the WSN approach makes it possible to easily represent the time series of the mean SM patterns/trends for a small area that match a required spatial resolution needed for modeling studies.

SM and WP surfaces (1-m cell size) were generated to illustrate the average spatial and temporal variability of these two parameters. The surfaces were built using the OK method. Along with the interpolated surface, elevation contours were generated from a 2-m resolution LIDAR raster, in order to complement the surface analysis by providing elevation input. The interpolation boundary was defined based on the area extent (approximately 15,000 m^2^) where the nodes are located. The highest and lowest elevations within the site are 365 and 346 m above mean sea level (m.a.m.s.l.), respectively.

**Figure 12:**
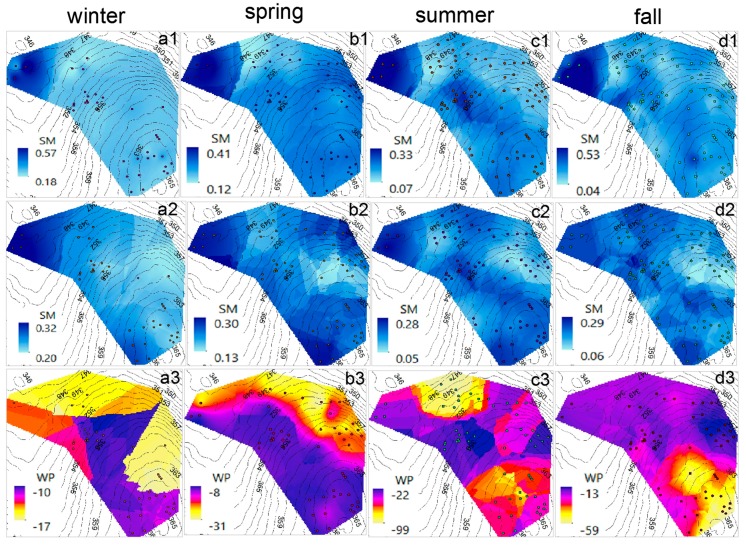
Interpolated surfaces (Kriging method) showing the average seasonal variation in volumetric soil moisture (SM) and soil water potential (WP), based on data retrieved from 2010 to 2016. (**a**) winter average (December–February): (**a1**) SM at 10 cm; (**a2**) SM at 30 cm; (**a3**) WP at 30 cm; (**b**) spring average (March–May): (**b1**) SM at 10 cm; (**b2**) SM at 30 cm; (**b3**) WP at 30 cm; (**c**) summer average (June–August): (**c1**) SM at 10 cm; (**c2**) SM at 30 cm; (**c3**) WP at 30 cm; (**d**) fall average (September–November): (**d1**) SM at 10 cm; (**d2**) SM at 30 cm; (**d3**) WP at 30 cm. SM is expressed in m^3^·m^−3^. WP is expressed in kPa. The elevation contours are expressed in m. The dots represent the nodes from which the data was retrieved.

Figure 12 shows the average-seasonal SM (at 10 and 30 cm) and WP (at 30 cm) surfaces from 2010 to 2016. Overall, it is noticed that the higher SM area is located in the lower part (at 346 m.a.m.s.l.), within a flatter region near a pond. Also, it is observed that SM, regardless of the elevation, is more homogeneous during winter than in the other seasons, which might be caused by snow accumulation and melting over the winter. This is more evident in the average winter SM at 10 cm (Figure 12a1), since the shallow soil is more influenced by the varying climatic conditions than the deeper soil [90].

Another noticeable fact is that the average variation in SM is higher at 10 cm than at 30 cm, suggesting that the longer travel time to deeper soil reduces the spatial variability of SM. SM is lower during the summer than in the other seasons, which is consistent with the recorded daily rainfall data at the Pittsburgh Airport Meteorological Station [91] for the 2010–2016 period, where there is a dry period between the end of the spring and summer months (i.e., May–September). Finally, based on the SM surfaces from summer and fall (Figure 12c1,c2,d1,d2), there seems to exist a water pathway (darker color in the surface) from the highest elevation to the lower part of the area, located at the right side of the SM surface. This pathway might be explained by the natural surface and subsurface water movement towards the creek located to the south of the study area which, in turn, drains into the pond (immediately downstream of the region with higher SM). Topography showed stronger influence on SM during the winter. Regarding WP, the interpolated surfaces show a variable behavior from one season to another, but WP is mostly higher in the regions with higher elevations, even though there are some lower regions with higher WP.

**Figure 13:**
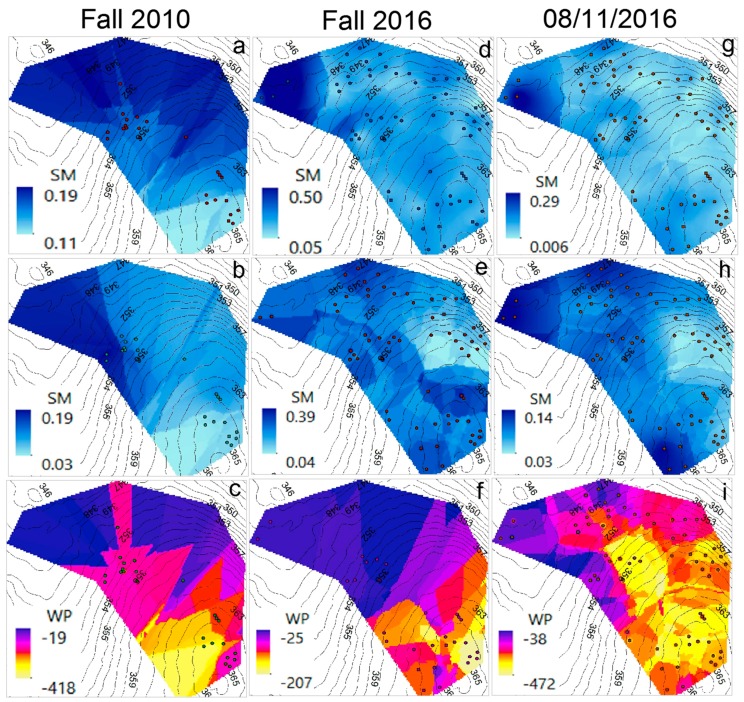
Interpolated surfaces (Kriging method) showing a comparison between the average fall (September–November) SM and WP of 2010 and 2016, and average SM and WP on 11 August 2016 (08/11/2016). (**a**) SM at 10 cm (fall 2010); (**b**) SM at 30 cm (fall 2010); (**c**) WP at 30 cm (fall 2010); (**d**) SM at 10 cm (fall 2016); (**e**) SM at 30 cm (fall 2016); (**f**) WP at 30 cm (fall 2016); (**g**) SM at 10 cm (11 August 2016); (**h**) SM at 30 cm (11 August 2016); (**i**) WP at 30 cm (11 August 2016). SM is expressed in m^3^·m^−3^. WP is expressed in kPa. The elevation contours are expressed in m. The dots represent the nodes from which the data was retrieved

Figure 13 illustrates the improvement achieved by the network expansion. In highly complex and heterogeneous environments, the amount and quality of data is proportional to the amount of extractable knowledge [92]. SM and WP interpolated surfaces were created for the average fall conditions in 2010 and 2016. Additional surfaces were generated for the average of 11 August 2016, which is the day with the highest recorded alive nodes (102 nodes, including the relays). In general, these surfaces show similar patterns for each corresponding variable (i.e., SM at 10 cm and 30 cm and WP at 30 cm). However, the 2016 network size, with less scattering in the node locations, provides better estimations than the 2010 network size. It is observed that the patterns for fall 2016 and 11 August 2016 are more similar to each other than the patterns for fall 2010 (see Figure 13). This indicates that the higher node density in 2016 provides more detailed insights of the temporal and spatial variability of SM and WP. Overall, the analysis has shown the applicability of WSNs for short and long term hydrological patterns characterization at the catchment scale, for steep-forested environments.

In addition, Table 4 shows the RMSE obtained from the OK interpolation for the scenarios presented in Figure 12 and Figure 13. The RMSE has the same units as the analyzed variable (i.e., SM or WP). The number of nodes from which the data was extracted, along with the maximum and minimum SM and WP values are also included. In terms of the SM, it is observed that RMSE is lower at 30 cm than at 10 cm, which is consistent with what has been shown before (i.e., that the SM is much more variable in the near-surface soil than in the deeper soil). In the case of the different average seasonal conditions, even with a different density of nodes within the site, the RMSE did not experience a significant change, thus showing the robustness of the OK interpolation method.

The lowest RMSE in SM, for both depths, obtained for 11 August 2016, suggests that a shorter period of time and a higher density of nodes reduce the uncertainty of the SM estimation. The estimated RMSE in the WP surfaces showed more variability than in the case of SM, mostly due to larger differences in the WP ranges for the analyzed conditions. However, if considering the error as a percentage of the range, the 11 August 2016 and fall 2016 average scenarios have the lowest percentages, 0.061% and 0.098%, respectively. In summary, geostatistical tools such as the OK interpolation constitute an important complement to WSNs for environmental monitoring purposes, especially when it is intent to estimate the spatiotemporal behavior of hydrological parameters.

### 3.5. WSN Challenges and Utility in Hydrology

There are several challenges faced in outdoor environmental monitoring WSN deployments, including power management, node maintenance, network scaling, heterogeneous deployment, and overall network cost [93].

#### 3.5.1. Power Management

It is of critical importance to maintain a constant power supply to the WSN nodes to ensure data collection and communication within the network. By far, the most common maintenance task is the replacement of batteries. Rechargeable nickel-metal hydride (NiMH) AA batteries were selected for powering the wireless motes as an environmentally friendly and cost-conscience means of maintaining the frequent battery changes of the network. Other alternatives such as the use of lithium-ion polymer battery (LiPo) were discarded due to budget constraints and the existing investment on a large number of AA (NiMH) batteries and chargers. Previous studies that analyzed the power efficiency of WSN motes using AA (NiMH) batteries showed that the expected autonomy of individual nodes is between 48 days [94] and 58 days [32]. More information about the energy profile for WSN nodes is available in [95]. The use of solar panels has been considered, but, with the dense forestation surrounding the majority of the network, it did not appear to have a sufficient return on investment; although, it might be suitable for other locations with more exposure to direct sunlight or during the winter months following tree leaf senescence.

Despite the benefits of rechargeable batteries, some drawbacks exist. Following a recharge, the NiMH batteries may have a significantly higher voltage (e.g., 1.4 V). This leads to circumstances where the combined voltage of three NiMH batteries (i.e., 4.2 V) is significantly greater than the recommended safe operating voltage for the wireless motes (i.e., 3.3 V). Also, issues with irregular charging voltages were found in the NiMH batteries, which were sorted based on the recommended screening process described in [32]. In order to maximize the life span of each relay node for each battery cycle, it is recommended using D batteries that have a capacity of about 10,000 mAh or more.

Over the span of the project, two sorting strategies were used for the batteries: full and partial sorting. In the full sorting strategy, before a maintenance event, all recharged batteries are sorted by their standing voltage from low to high. Replacement batteries are then chosen as consecutive groups of three from the sorted group. In the partial sorting strategy, batteries are grouped based their standing voltage into bins (e.g., 1.25–1.30 V). Replacement batteries for a single mote are then taken from the same bin. In this method, only voltage bins with an adequate number of batteries are used, which often leads to unused batteries in bins with only one or two batteries. In this regard, the full sorting strategy is slightly better; however, it is more time consuming. As indicated in Figure 16 of [32], node battery life throughout the network improved following the adoption of a battery sorting strategy.

Another method for improving the battery life of wireless motes is to reduce its number of transmissions. This is due to the high-energy costs of transmitting wireless data [94,96]. During the first years of this project, each node had a sampling rate of 15 min. This was a trade-off between the desired sub-hourly temporal resolution of the environmental data, the expected battery life for each power cycle of the motes’ batteries, and the poor packet reception rate of the network, which was around 50% during the first years of deployment. With recent versions of the WSN protocol, the packet reception rate has significantly improved to over 90% [32]. In addition, the new WSN protocol allows for the customization of network parameters for individual nodes according to their intended use. In order to reduce power consumption, the sampling rate of relay nodes (and some sensor nodes) was lengthened to 30 min. At the same time, to address measurement noise, the sampling rate for the sap flow nodes was shortened to 10 min. The increased sampling rate of the sap flow nodes was not a concern for battery life, as these nodes are powered by the 12 V lead-acid battery.

#### 3.5.2. Node Maintenance

The maintenance of the data collection equipment depends on knowing the status of each individual node or data logger. However, the data loggers used in this study are not available on-line and therefore it is not possible to monitor the data collected with them in real time. In addition, in order to collect the data, the researcher needs to commute to the location where the data logger is located. There are some disadvantages with this approach. First, if a wire is loose, then data from one or several sensors attached to the data logger is lost. Second, if the batteries are depleted, then the data logger stops working. Third, there is no way to be aware of those issues until the data is downloaded and examined. Lastly, downloading the data from a data logger is time consuming and does not scale to the case of several locations because every location has to be downloaded independently. Also, the data for a single data logger generates a number of separate files (i.e., one at each location and time of downloading) that require further processing before analysis—as is the case for the Decagon Devices EM50 data logger used in this study. On the other hand, the data collected from our WSN is stored directly and automatically in a relational database that is available through a web-based integrated network and data management system for heterogeneous WSN site called INDAMS [97] for online monitoring.

One way to reduce the need for node maintenance is by using enclosures of high quality, even though they tend to be more expensive, their associated costs pays off in the long run as they are more resistant to environmental damage, less prone to water intrusion, easier to open and close, and, therefore, easier to maintain. In addition, high quality enclosures keep the sensing and communication equipment, and the batteries safer.

#### 3.5.3. Network Routing and Scaling

In multi-hop large-scale WSN networking, the routing protocol plays an essential role for reliably collecting sensor data in real time. While WSN deployments appear promising due to the limitations of traditional data logging methods [98], the WSN scalability has proven to be a bottleneck in early studies. An increased network size introduces more data traffic, collisions and congestion in the network, resulting in network performance degradation. To mitigate this problem, starting from the summer of 2014, the ASWP network has adopted CTP + EER routing, which significantly reduces the workload of motes along efficient routes and thus extends the WSN lifetime.

During the network expansion, from 52 nodes in 2014, to 88 nodes in 2015, and finally to 104 nodes in 2016, the network performance has not been noticeably influenced while operating CTP + EER. The packet reception rate (PRR) of the network remains above 96%. The average packet path length is 3.95 hops during the 52-node network, 4.76 hops during the 88-node network, and 4.73 hops during the 104-node network. In the summer of 2015, both the width and length of the WSN deployment was expanded, which caused the increase of the average path length. In the summer of 2016, the major network change was its density, which caused a slight reduction in the average path length and PRR. This result demonstrates that with the proper routing protocol, the network is able to maintain high levels of performance over various deployment scales.

#### 3.5.4. Heterogeneous Mote Reprogramming

The ASWP WSN deployment consists of three different mote platforms as well as multiple application versions (corresponding to various external sensors attached to individual motes). As an exploratory and evolving WSN deployment, the network application needs to be updated frequently to test new protocols and parameter configurations. Over-the-air reprogramming approaches become a natural choice since manually reprogramming the motes is cumbersome. The heterogeneous nature of the developed WSN with motes operating in LPL makes the existing reprogramming tools infeasible [52,53].

**Figure 14:**
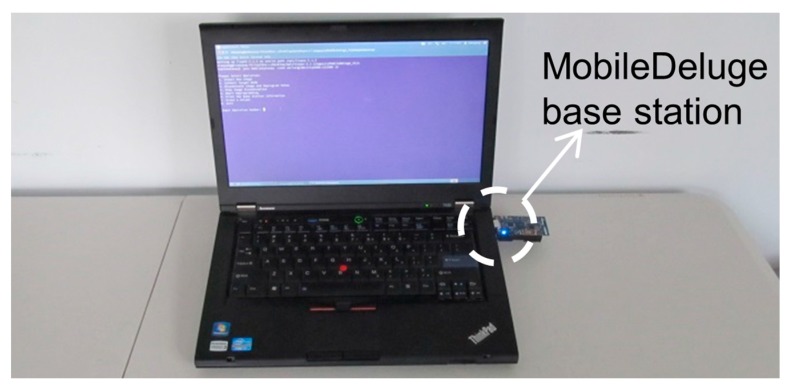
MobileDeluge, a hand-held mobile mote reprogramming tool.

Our developed MobileDeluge [52] is a novel hand-held mobile over-the-air mote reprogramming tool for outdoor WSN deployments (Figure 14). MobileDeluge builds a new control layer on top of Deluge [99]. It enables and disables Deluge services on demand, allowing for the selection of a subset of motes as targets when initiating a reprograming task. It then disables LPL in the targets for fast dissemination of the new application image, which usually consists of thousands of packets. The targets are also configured in a different radio channel to avoid interference with the rest of the network. MobileDeluge currently works with the motes within a one-hop range to avoid forwarding a bulk code image over intermediate nodes for mote energy conservation. Please see [52] (and the references herein) for more details.

MobileDeluge has significantly reduced the time and labor required to update the application in the outdoor WSN testbed. The manual reprogramming procedure would consist of getting the enclosure from the tree, opening the box, attaching the mote to the laptop and uploading the new application. For example, it usually takes a few days to reprogram the whole ASWP testbed (i.e., 104 motes). With MobileDeluge, in contrast, the reprogramming can be finished within one afternoon.

#### 3.5.5. Network Costs

The 52-node MICAz and IRIS network at the end of 2014 had a cost of $31,500 for the wireless motes, gateway, sensors, and other peripherals [32]. The CM5000-SMA (TelosB) mote includes built-in humidity and temperature sensors and does not require the use of an acquisition board for relay nodes as opposed to the MICAz or IRIS motes that use the MDA300 acquisition board ($179). Therefore, the adoption of the TelosB motes has significantly reduced the cost of relay nodes, from $330 (in the MICAz network) to $164, despite the TelosB ($110) being slightly more expensive than the MICAz or IRIS motes (both models about $99 each). These savings are also found for the soil sensor nodes (from $664 to $480), mainly due to the deployment of our inexpensive ($13) designed sensor boards (with 5 V voltage booster) instead of the MDA300, despite the increased cost for the MPS-2 (compared to the MPS-1) sensor. A new sap flow box design, which replaced the MDA300 by our sensor board ($9) without the 5 V voltage booster and does not require the use of AA or D batteries, has further contributed to the cost savings (from $464 to $257). The cost of the expanded 104-node (27 MICAz, 32 IRIS and 45 TelosB motes) network is approximately $50,000. Table 5 shows the distribution of sensors for each type of node.

For the sake of comparison, a Decagon Devices EM50 data logger costs about $476 and is roughly equivalent to our WSN soil sensor node ($177 without the sensors) in terms of its capability to host external sensors.

### 3.6. Lessons Learned with Sap Flow

Low-cost wireless sap flow monitoring is a challenge for environmental research. The delicate nature of the thermal dissipation sap flow sensor, not often surviving more than a single season, and the price of the commercial sap flow sensor, which is too high for large deployments with tight budgets, lead researchers to building their own sensors. While cost effective [39], there are challenges to manufacturing working sensors, which require a good deal of patience and careful attention to detail. Once manufactured, sensors should undergo calibration to account for slight variations in workmanship and care must be taken during transport and installation, during which time the heating filament can be easily damaged. The cost effectiveness of these self-made sensors outreaches the drawbacks of their tedious manufacturing and delicate installation.

There is also the issue regarding the integration of sap flow sensors into WSNs. Early WSN sap flow studies were based on experimental hardware burdened with power limitations and software development issues [28,100]. These days, good wireless implementations are becoming more and more ubiquitous and more seamless in terms of user experience.

## 4. Conclusions

The environmental data collected with the WSN nodes were found to be similar to the data collected from the Decagon Devices Em50 data logger in terms of quality. However, the WSN nodes overcome some important limitations of traditional data loggers at a significantly lower cost. For instance, the data readings from the WSN nodes are automatically collected and stored in a relational database system, therefore all the environmental data are saved in a unified and integrated repository, eliminating the need to manually download data at each location. In addition, the status of individual nodes is available in a web-based integrated network and data management system developed for heterogeneous WSN site called INDAMS.

This study has shown an effective application of WSNs to determine and explain spatiotemporal hydrological patterns. A specially designed sensor board provides stable excitation voltage for analog and digital sensors at only approximately 6% of the cost of the MDA300 acquisition board. MPS-2 sampling synchronization issues on sensor motes were solved with our driver software developed in TinyOS. Our exploratory study demonstrates how the innovative WSN routing protocol CTP + EER and the over-the-air reprogramming tool MobileDeluge can overcome the challenges of heterogeneous and large-scale multi-hop WSN for outdoor environmental morning. In particular, this study has presented the first of its kind comprehensive data analyses for the WSN monitored hydrological variables including soil temperature, WP, SM and sap flow. Two PTF parameters that are utilized by hydrologic models to predict soil water retention properties (i.e., the Clapp-Hornberger equation and the van Genuchten equation) were estimated with the retrieved SM and WP data with a high goodness-of-fit (i.e., NSE greater than 0.80). The improved installation design of the sap flow sensors allowed for the retrieval of high-quality data, which later were filtered using a robust weighted local regression to smooth the data without being affected by the outliers. At the same time, these sap flow data were used to estimate transpiration rates, which were highly consistent with previous studies in sites with similar geographic and climatic characteristics. The estimation was also consistent with the local measured data (meteorological stations). Moreover, a spatial analysis was performed to show the variability of SM and WP, which showed the applicability of WSNs for short and long term hydrological patterns characterization in a catchment scale in steep-forested environments.

It has also been shown that “out of site” procedures, such as sensor calibration methodologies and adequate data processing, provided a fundamental added value to the field work. Finally, despite the tremendous challenges posed by outdoor WSN deployments, including power management, node maintenance, routing scale, heterogeneous deployment, and overall network cost, the wireless sensor network approach (e.g., protocols, sensors, deployment tool, and acquisition) presented in this study has proved to be an effective (in terms of the data quantity and quality) and low-cost alternative for environmental monitoring. This helps pave the way to larger scale outdoor WSN developments in the future in order to ultimately study and answer the fundamental science questions for quantifying sub-grid heterogeneity and in understanding hydrologic parameters.

Future work should also consider continuing exploring materials and methods to lower the cost of the network without reducing the data quality and other complementary strategies such as the optimization of battery usage.

## Figures and Tables

**Table 1:** Soil parameter calibration results for the Clapp and Hornberger, and Van Genuchten PTFs.

Location	Depth (cm)	*θ_s_*	b	*ψ_s_* (kPa)	NSE (Clapp-Hornberger)	n	*α*	NSE (Van Genuchten)
DL1	10	0.32	9.63	0.045	0.914	1.093	62.34	0.929
DL1	30	0.44	10.69	0.00066	0.800	1.081	10814.0	0.821
DL2	10	0.31	4.49	0.658	0.921	1.215	1.808	0.922
DL2	30	0.42	5.87	0.521	0.801	1.154	3.51	0.812

**Table 2:** Comparison of silver maple (Acer saccharinum) sapwood area from [66] measurements and Equation (6) estimations in site 2 of the ASWP network.

Node	d (cm) ^a^	$A_{S}$ (%) ^b^	$A_{total}$ (cm^2^) ^c^	${\hat{A}}_{S}$ (cm^2^) ^d^	${\hat{A}}_{S}$ (cm^2^) ^e^
2045	34	77.4	839	650	700
2055	30.7	70	682	478	601
2065	31.5	76.7	719	552	633
2095	41.2	81.7	1250	1020	954
2115	24.3	83.1	415	345	394

^a^ based on hand measurements made in 2010; ^b^ based on the average of three core samples taken in 2010; ^c^ assumes 0.64 cm bark thickness; ^d^ based on the estimated percentage of sapwood area times the total trunk cross-sectional area; ^e^ based on the regression equation of [61] where $B_{0}$ = 2.052 and $B_{1}$ = 1.654.

**Table 3:** A_S_/A_G_ calculations based on the field survey within the three survey regions in site 2 of the ASWP site.

Survey Regions	$\sum {\hat{A}}_{S}$ (m^2^) ^a^	$A_{G}$ (ha) ^b^	$A_{S}/A_{G}$ (m^2^/ha)
2134	0.375	0.0292	12.87
2084	0.38	0.0391	9.73
2014	0.784	0.0593	13.23

^a^ sum of ${\hat{A}}_{s}$ values within each survey region, based on hand measurements taken at a height of 1.37 m in 2016 and Equation (6), where $B_{0}$ = 2.052 and $B_{1}$ = 1.654 [61]; ^b^ based on Thiessen polygon areas (Figure 6).

**Table 4:** RMSE of the interpolated surfaces.

Interpolated Surface	SM (m^3^·m^−3^) at 10 cm				SM (m^3^·m^−3^) at 30 cm				WP (kPa) at 30 cm			
	# Nodes	Max SM	Min SM	RMSE	# Nodes	Max SM	Min SM	RMSE	# Nodes	Max WP	Min WP	RMSE
Winter 2010–2016	38	0.570	0.180	0.061	38	0.320	0.200	0.030	37	−10	−17	2.12
Spring 2010–2016	55	0.410	0.120	0.062	57	0.300	0.130	0.048	51	−8	−31	4.94
Summer 2010–2016	71	0.330	0.070	0.061	72	0.280	0.050	0.052	59	−22	−99	17.46
Fall 2010–2016	72	0.530	0.040	0.063	72	0.290	0.060	0.051	57	−13	−59	6.01
Fall 2010	24	0.190	0.110	0.059	23	0.190	0.030	0.058	24	−19	−418	41.91
Fall 2016	72	0.500	0.050	0.026	72	0.390	0.040	0.057	69	−25	−207	17.88
8/11/2016	74	0.290	0.006	0.031	74	0.140	0.030	0.024	74	−38	−472	26.53

**Table 5:** Distribution of nodes by type of application.

App Type	Number
Relays	27
Soil Moisture Water Potential (EC-5 × 2, MPS-1 × 1)	31
Soil Moisture Water Potential (EC-5 × 2, MPS-2 × 1)	36
Sap Flow	10
